# A qualitative study of experiences of NHS mental healthcare workers during the Covid-19 pandemic

**DOI:** 10.1186/s12888-021-03261-8

**Published:** 2021-05-12

**Authors:** Elisa Liberati, Natalie Richards, Janet Willars, David Scott, Nicola Boydell, Jennie Parker, Vanessa Pinfold, Graham Martin, Mary Dixon-Woods, Peter B. Jones

**Affiliations:** 1 Department of Public Health and Primary Care, THIS Institute (The Healthcare Improvement Studies Institute), University of Cambridge, Cambridge, UK; 2 Department of Health Sciences, University of Leicester, Leicester, UK; 3 Population Health and Genomics, University of Dundee, Dundee, UK; 4 Centre for Biomedicine Self and Society, Usher Institute, University of Edinburgh, Edinburgh, UK; 5 McPin Foundation, London, SE1 4YR UK; 6 Department of Psychiatry, Cambridgeshire & Peterborough NHS Foundation Trust, University of Cambridge, Cambridge, CB2 0SZ UK

**Keywords:** Mental health services, Staff, Healthcare workers, Covid-19, Qualitative

## Abstract

**Background:**

The Covid-19 pandemic has imposed extraordinary strains on healthcare workers. But, in contrast with acute settings, relatively little attention has been given to those who work in mental health settings. We aimed to characterise the experiences of those working in English NHS secondary mental health services during the first wave of the pandemic.

**Methods:**

The design was a qualitative interview-based study. We conducted semi-structured, remote (telephone or online) interviews with 35 members of staff from NHS secondary (inpatient and community) mental health services in England. Analysis was based on the constant comparative method.

**Results:**

Participants reported wide-ranging changes in the organisation of secondary mental health care and the nature of work in response to the pandemic, including pausing of all services deemed to be “non-essential”, deployment of staff across services to new and unfamiliar roles, and moves to remote working. The quality of participants’ working life was impaired by increasing levels of daily challenge associated with trying to provide care in trying and constrained circumstances, the problems of forging new ways of working remotely, and constraints on ability to access informal support. Participants were confronted with difficult dilemmas relating to clinical decision-making, prioritisation of care, and compromises in ability to perform the therapeutic function of their roles. Other dilemmas centred on trying to balance the risks of controlling infection with the need for human contact. Many reported features of moral injury linked to their perceived failures in providing the quality or level of care that they felt service users needed. They sometimes sought to compensate for deficits in care through increased advocacy, taking on additional tasks, or making exceptions, but this led to further personal strain. Many experienced feelings of grief, helplessness, isolation, distress, and burnout. These problems were compounded by sometimes poor communication about service changes and by staff feeling that they could not take time off because of the potential impact on others. Some reported feeling poorly supported by organisations.

**Conclusions:**

Mental health workers faced multiple adversities during the pandemic that were highly consequential for their wellbeing. These findings can help in identifying targets for support.

## Background

Recognition that the physical and mental wellbeing of healthcare workers is critical to high quality, safe and empathic healthcare is increasingly growing [1]. Supporting wellbeing is now understood as an ethical imperative and employer responsibility [2, 3]. It is also important in reducing sick leave and staff turnover, and thus in securing the sustainability of health systems [4, 5]. However, a systematic review found that healthcare workers are at especially high risk of mental health difficulties (including anxiety and depressive symptoms) during viral epidemic outbreaks [2], a problem that has been vividly highlighted during the Covid-19 pandemic [6].

On 23rd March 2020, the UK government implemented lockdown measures in an attempt to reduce the transmission of the SARS-CoV-2 virus and halt the pandemic of Covid-19. People were instructed to stay at home and avoid all ‘non-essential’ contact with others outside of their household. The introduction of these measures had a profound impact on the healthcare system while simultaneously creating a range of extraordinary strains on healthcare workers. As well as exposure to a potentially deadly infection that could affect them directly or be transmitted to their families, many staff were routinely faced with unprecedented situations, deaths of those they were caring for, and having to make highly consequential decisions under severe pressure [7].

The need to attend to the mental health of healthcare workers was raised early in the pandemic [8, 9]. Much of the focus of attention and research on the impacts of the pandemic to date has been on those working in acute medical settings, including, for example, intensive care units [7, 10]. The difficulties confronted by those working in mental healthcare settings, both in-patient and in the community, have been less well scrutinised and understood, but are no less important. Working practices have changed dramatically [11–13] while population need and referrals to care are likely to continue to increase. In this article, we respond to this void in the literature, reporting an interview-based qualitative study that seeks to characterise the challenges faced by those working in NHS mental health settings during the first wave of the Covid-19 pandemic in 2020.

## Methods

The study we report here is one element of a larger project involving service users, carers, and NHS staff in England. This larger project was designed and developed with six experts by experience (three service users and three carers) and a peer researcher from the McPin Foundation, a mental health research charity.

Between June and August 2020, we undertook a qualitative study involving remote interviews with 69 people, including 24 people with mental health difficulties, 10 carers, and 35 point-of-care staff working in NHS secondary mental health services. During this period many inpatient mental health units had reduced their bed capacity and raised their thresholds for admissions in order to adopt strict infection control measures. Consultant psychiatrists and other clinical leaders were often purposefully largely absent from wards due to infection control policies, and the system was adjusting to the realisation that staff moving between wards presented a considerable risk of transmitting infection between service users and colleagues. Community-based teams had pivoted away from face-to-face assessment and treatments towards remote methods involving a variety of previously unfamiliar video-conferencing platforms. New methods of working disrupted day-to-day contacts between clinical, management and administrative staff. The analysis we present here focuses on the 35 clinical staff interviews undertaken in the context of these changes; data from the other interviews are reported elsewhere [11].

We adopted a purposive sampling strategy [14], not aiming to achieve statistical representation of the population, but to identify a variety of experiences related to our research questions. As data collection and analysis progressed in parallel, the size of the sample was adapted to the variety of experiences captured, in line with the principle of information power [15]. Multiple channels were used to publicise the staff study, including the networks of the researchers and specialty clinical networks. Some participants engaged in response to information circulated through dedicated networks, and other participants become involved as a result of colleagues or friends alerting them to the study (a technique known as snowball sampling).

Invitations were circulated with an expression of interest form, and people who wished to take part were asked to use the form to indicate their ethnicity, the gender they identified with, and first half of their postcode. We then reviewed the characteristics of those who had completed a form to optimise diversity in the sample, particularly in relation to minority background, and to achieve balanced gender representation and widespread geographic cover in England (Table 1).

**Table 1:** Participants’ demographic information

	Staff demographic information
Gender	• 19 Female • 11 Male • 5 people did not provide this information
Ethnicity	• 24 White • 3 Asian • 2 Mixed ethnicity • 1 from ‘any other ethnic group’ • 5 people did not provide this information
Region	• 4 North East • 7 North West • 2 East Midlands • 5 West Midlands • 4 Greater London • 2 East of England • 3 South East • 3 South West • 5 people did not provide this information
Job role	• 17 Psychiatrists (including 13 trainees and 4 consultants) • 10 Mental health nurses (including care coordinators, clinical leads, non-clinical prescribers) • 5 Psychotherapists (including CBT therapists and systemic family therapists) • 3 Clinical psychologists
Services	• Community Mental Health Teams (CMHT) • Early intervention for psychosis (EIP) • Crisis Teams • Acute hospital wards • Secure Forensic services

Potential participants were contacted by the researchers via phone or email, depending on their preferred contact method, and were provided with a link to register on Thiscovery, a secure online research platform developed by THIS Institute according to the AA Web Content Accessibility Guidelines to ensure that it meets accessibility standards.

To comply with the UK lockdown regulations, all interviews were conducted remotely. Participants could choose between being interviewed over the phone (21 staff) or using online video-supported secure software (14 staff). JW, NR and EL conducted the interviews using a topic guide which covered a range of topics but was deliberately non-directive to allow participants to discuss areas they perceived as relevant. Interviews lasted between 30 and 95 min.

Interview audio files were securely transferred to a third-party transcription service subject to the University of Cambridge data protection regulations. Analysis of anonymised interview transcripts was based on the constant comparative method [16]. The coding scheme was developed based on a subset of initial interviews. The initial codes were revised, expanded and collapsed as analysis progressed, and through whole team discussions. Codes were then were organised into categories in a thematised coding scheme. DS, NR, NB, JW and EL analysed staff interviews. Data was processed using NVIVO software by four coders (three females, NR, NB and EL, and one male, DS). Some interview excerpts were edited to remove information that might reveal the identity of participants.

Ethical approval for the study was obtained from the University of Cambridge Psychology Ethics Committee on 15 June 2020. All participants were provided with information about the study. Electronic, written informed consent was obtained from all participants. We were guided by the Standards for Reporting Qualitative Research recommendations [17].

## Results

Participants in the study included psychiatrists (trainees and consultants), care coordinators, mental health nurses, clinical psychologists and psychotherapists (Table 1). Their experiences of working during the pandemic varied according to service type, geographical location, and prevalence of Covid-19 infections, but all conveyed an overwhelming sense of systemic shock. Below, we give an account of participants’ descriptions of: the wide-ranging changes in services and the nature of work brought about by the response to the pandemic; the impacts of these changes on staff; the dilemmas and challenges they faced; and wellbeing and use of support.

### Changes in services and the nature of work

The most immediate consequence of the pandemic described by participants was the rapid re-organisation of both inpatient and community services to minimise risks of infection. Senior NHS leaders made rapid decisions to prioritise essential over non-essential services. These decisions were usually within an incident management team structure and were cascaded to service managers (who were generally not themselves part of the decision-making process) to operationalise and implement. Some previously in-person services changed to being provided remotely (e.g. by telephone and/or by video-conferencing); others were withdrawn or paused to preserve the functioning of services that were deemed to be essential, with risk assessments put in place to prioritise allocation of care.

*So the entire of the service was geared towards ( … ) the crisis part, and what we ended up doing was essentially traffic-lighting everyone, using the Red-Amber-Green rating, and that then would dictate how frequently a mental state was performed on risk. The focus of the organisation changed overnight pretty much.* (Trainee Psychiatrist, Community mental health service)

To ensure that services deemed essential were adequately staffed, some members of staff were redeployed to frontline mental health settings, often with very little notice and without consultation. Some staff were moved, for example, from community mental health services to inpatient wards. Some had to work from home. Consultants (senior doctors) were often asked to work remotely, in order to reduce the risk that they would become infected and leadership would be lost.

*The consultants are considered the most valuable because we’ve only got one responsible clinician per ward and if something happened to them, then the whole trust would be in trouble. ( … ) I know that our consultant doesn’t like it but they can understand that rationale and they’re still fully involved in every decision, ( … ) but they’re not allowed onto the ward.* (Staff nurse, Acute adult ward)

The changes arising from the reorganisation of services resulted in profound alterations in the work participants did and the nature of that work. Some described being redeployed to unfamiliar and more acute frontline mental health services, often involving increased exposure to individuals with Covid-19, and having to work in personal protective equipment (PPE). Where staff were not redeployed, many had to deal with rapid change to telephone working and service delivery, and later to video-conference methods; others were left uncertain about what was going to happen, day to day.

Many acute mental health hospital wards were reorganised in an attempt to maintain separation between COVID-positive and COVID-free service users as an infection control measure. Systematic assessments of personal infection risk were late in occurring, and even then there was variability about how rapidly and efficiently they happened. As a result, staff felt worried about working in situations that they considered to be unsafe (e.g. because of staff-to-staff and staff-to-service user contagion). Some mental health wards received testing equipment and PPE early on in lockdown, and were able to swiftly create appropriate isolation areas. In other cases, participants described a more chaotic scenario, where systems to contain viral spread were lacking, and social distancing measures were hindered by structural constraints. Some infection control arrangements were difficult to sustain (e.g. because of demand for bed space) or were seen to introduce other challenges.

*A patient had to get a chest x-ray, but in our ward [there are] low immunity patients, [and] if a patient goes to general medical ward then they are exposing [everyone] to COVID. ( … ) So we had to isolate the patient for seven days … but our patients’ mental health is already fragile and keeping them in isolation they wouldn’t be able to attend the ward programmes, for example, the groups that we’re doing, sit and have a meal with all the other patients. ( … ) So [the patient] didn’t get better at all.* (Trainee Psychiatrist, Inpatient ward)

### Impacts of Covid-19-related service changes on staff

Changes to services introduced in response to the pandemic meant sudden and unsettling alterations to what work was prioritised and how it was done, as well as multiple and ongoing uncertainties and absences of clarity. Individuals working in inpatient settings on the frontline (mostly trainee doctors and nurses) reported being faced with greater responsibilities and longer shifts to cover for colleagues who were shielding, self-isolating, absent with caring responsibilities or otherwise absent. They reported an expectation that they would go above and beyond in their role in order to keep things afloat, which, coupled with little recognition for their work, led to increased stress. Pressures on psychiatry trainees (especially those working in hospitals) were compounded by the removal of consultants from face-to-face patient contact, which resulted in increased complexity and responsibility for trainees. Psychiatric consultations and ward rounds were sometimes conducted through a hybrid system involving trainee doctors and nurses being present in-person to do their own roles as well as having to represent the consultant’s authority, responsibility and perceived power, while consultants attended remotely.

*Our consultant would be there via Teams on a laptop. And we’d have to point the laptop at the [service user], and then myself or one of the other doctors would be asking questions, and the consultant could take over and ask whatever questions they wanted … The consultant was probably heavily relying on the nurses and the doctors there, rather than himself, to look at smaller behaviours … little things like facial reactions, body language, things like that.* (Nurse, Acute adult ward)

Trainee psychiatrists reported that tensions were high within inpatient settings due to the increased restrictions involved in infection control. For example, service users were not able to go outside for exercise or smoking breaks, and staff perceived that these restrictions and other pressures associated with the pandemic sometimes led to incidents that they saw as verbally or physically intimidating. Further, because consultants were not visible on the wards, psychiatry trainees reported a need to act at or beyond their own perceived authority. For instance, they had to address service user questions about treatment decisions and to manage other interactions that they found challenging. While participants saw the behaviours as understandable given the situational strains on service users, they reported that anticipating and dealing with incidents was sometimes stressful.

*It was a lot, like, people felt they were being treated like children. And so, if you are psychotic, a bit more agitated, there was the whole manic season during spring, so April, May there's always a spike in mania. So, can you imagine this really high agitated people in a small contained room, it was just … it was really quick to get into a fight or an argument. And then staff were like, okay, I need to keep you in this bedroom.* (Trainee psychiatrist, Inpatient ward)

*Because when there isn’t a representative senior, like a consultant, patients become very upset and they become agitated. And they appeal their sections under the Mental Health Act and they’re dissatisfied with their care and then they get aggressive.’* (Trainee Psychiatrist, Acute adult ward)

Staff who moved to remote working based at home encountered different challenges. The boundaries separating home and work became blurred, sometimes resulting in a pervasive sense that people were always ‘at work’ or ‘on call’. Breaks between consultations (which, before the pandemic, were enabled by travelling time) were reduced or removed altogether, and often staff found themselves working longer hours to ‘compensate’ for what would have typically been commuting time to and from work. Several members of staff reported feeling guilty that they were working from home while their colleagues on the frontline were not coping well. Back-to-back appointments resulted in a loss of opportunity to process and debrief after consultations. Many participants reported symptoms of burnout and expressed concern that if the pandemic lasted much longer [as it has done] this would worsen, considerably.

*It’s really easy to work way above your hours because the computer and the phone is just always there. … Like, for example, today I started at seven, and before you know it... you might find yourself sat at the computer still at six … you just … kind of, lose a bit of track of time, and then you feel a bit guilty having a half an hour lunch break because you should be on the computer*. (CBT Therapist, Early intervention in psychosis service)

*… so, there was a clap every week for the NHS, and you, sort of, felt like a bit of a fraud really, ‘cause you thought well I’ve not really … I've not really contributed that much this week. Actually, what I was doing was staying at home and … writing reports and dialling into meetings where I could. Which felt very limited when … you hear some of the stories that went on in the acute setting* (Forensic psychiatrist, Secure hospital)

The new arrangements were particularly challenging for staff who shared living space with other people or had childcare responsibilities, especially when they were still expected to hold confidential and intense consultations with service users.

Interacting with colleagues remotely also presented a new set of challenges, especially linked to the deprivations of informal, unstructured conversation. Many reported that multidisciplinary team meetings to discuss cases were diminished in quality: the bounded nature of the meeting within a specific timeslot meant there was little opportunity to carry on discussions informally and learn with and from colleagues. The challenges associated with remote working often led to staff feeling isolated in their decision-making, and anxious about making the wrong decision about an individual’s care.

*We have our morning meetings, but when we’re in the office together we spend a lot of time discussing things, mulling things over, working things out with each other, and, yeah, we can pick up the phone, but it’s not the same. It’s not as readily available. So, the decisions are being made much more in isolation. So, there isn’t that check and balance.* (Senior care coordinator, Early intervention in psychosis service)

It was a difficult and isolating time for trainee doctors; they reported limited opportunity to interact with their peers during virtual teaching sessions. This decreased morale and meant trainees were afforded fewer opportunities to reflect on their practice with colleagues or improve decision-making.

*It’s been a more isolating time or period as a doctor, the camaraderie that’s normally there with junior doctors has gone entirely, because there’s no gatherings for teaching, it’s all virtual and anonymous, black circles with initials. And I find that sad, I find that quite difficult. And I think quite often when you’re practising, you get a degree of validation from others, you can perhaps measure yourself against others a little bit. Not in a competitive way, in a sense that you get a feeling that you’re doing things right or in line with others. Whereas, that’s gone, you don’t see people.* (Trainee psychiatrist, Older adults CMHT)

Formal clinical supervision usually remained accessible but staff suffered from losing the informal support that used to be available in a shared office or clinical space. Because interactions were formalised through online meetings, some participants missed the opportunity to engage in light-hearted conversations that could create social bonds and act as tacit but powerful support. Informal interactions that facilitated ‘checking in’ on colleagues and their concerns were also lost.

*Informal supervision, hearing conversations, ( … ) all that informal stuff I think actually really keeps things much safer. ( … ) There's something about people watching out for one another ( … ) If you're really stressed and you're having a really bad day, you would turn to your neighbour and talk to them. ( … ) Or if something really dodgy was going on, someone would go to the manager and say, ‘Oh, do you know what’. None of that will get seen because it's all happening at home. So, for me, that's a worry.* (Family therapist, Early intervention in psychosis service)

*[ … ] it’s the sort of kind of informal contact that you get through just being in an environment and kind of moving through them and seeing people that can be very important and that it, yes, it’s that sort of informal corridor conversations, yes, chats in the kitchen, there’s probably more value in that than we realise and now everything is sort of scheduled and kind of formalised. Yes, so there is a loss with that, I think.* (Clinical psychologist, Early intervention in psychosis service)

### Dilemmas and challenges

Participants described being confronted by multiple challenges and dilemmas arising from the pandemic. Some of these centred on their perceived inability to care for people in the way they felt would serve their needs, or providing care that they perceived as sub-standard and that didn’t “feel right”. For example, some staff members expressed frustration about having to make clinical decisions based on an assortment of contingent information sources, such as second-hand reports from colleagues and limited non-verbal information gathered through remote-access technologies.

*You feel more anxious because as a clinician you are used to doing face-to-face, you are doing this on the phone, you worry that you will miss something and that was a big anxiety, at least for me. … It took some time for me to adjust to it, to be fair. I felt quite uncomfortable with it. At the moment even, if you talk about it now, I still feel uncomfortable* (Trainee psychiatrist, Older adults CMHT)

*I don’t have as much information to make decisions so I’m questioning my decision-making more thoroughly. I’m frightened of making the wrong decision when I’m deciding whether somebody gets a service or doesn’t get a service. That’s quite problematic.* (Senior care coordinator, Early intervention in psychosis service)

Lack of availability of technical support such as imaging facilities also meant that staff were not always able to perform a holistic, mind-body assessment. Staff often expressed their concern and feelings of helplessness about delays in treatment.

*So, if I’ve not done the memory test … because none of the hospitals are operating on full capacity. In dementia we usually screen by doing brain scan, memory test and history, so history is fine, that I can do on the phone, but I can’t do memory assessment properly. I can’t get a brain scan done, so everything that was meant to be done was delayed. So, I think at least for me that’s quite detrimental to the patient*. (Trainee psychiatrist, Older adults CMHT)

Though generally accepting that the pandemic had forced difficult decisions, many participants reported great discomfort with how services had been prioritised, as well as the way competing demands on services that remained open were managed. In a bid to reduce the risk of Covid transmission, and ensure adequate staffing levels in inpatient settings, many secondary mental health services had either been forced to shut or were operating with tightened admission criteria. Some participants believed that higher thresholds for admission were at odds with the ethos and philosophy of their service; others felt that the prioritising of some services over others undermined the proactive and preventative work that is key to early intervention.

*I understand the need to protect the core service if you like, but it’s been hard to send mixed messages about our referral criteria. To other services, for a very long time, we’ve been saying, if you even suspect psychosis, talk to us, you know, we want to know about it, liaise with us. And then at the moment, people are referring to us, and we’re saying we won’t even assess a lot of those referrals. And referrers are confused by that, I think. And I can understand why.* (CBT therapist, Early intervention in psychosis service)

Many psychiatrists and care coordinators felt that the therapeutic function of their role was particularly compromised in favour of ‘case management’, pointing to the moral content of these prioritisation decisions. Staff felt guilty that they were not able to refer service users to the necessary specialist services because these were no longer operational, they described service users as being in a ‘holding pattern’, rather than being supported to make improvements.

*So, a referral for autism is non-essential, a referral for ADHD is non-essential, a referral for psychological intervention of any kind, be it CBT [cognitive behavioural therapy], DBT [dialectical behavior therapy], IPT [interpersonal psychotherapy], any of the psychotic interventions is deemed non-essential. … It is purely crisis services and eating disorders that have been prioritised.* (Trainee psychiatrist, Community mental health service)

*The care coordinators who generally offer behavioural family therapy were no longer expected to offer it, it wasn't thought of as their priority of work.* (Family psychotherapist, Early intervention in psychosis service)

A distinctive feature of the accounts of participants was the evidence of moral injury linked to staff feeling that they were letting service users down and that people were suffering deeply as a result. Participants reported feelings of deep dissatisfaction with the support they were able to provide for service users during the pandemic. One worry was that individuals with mental health difficulties would be left feeling isolated and without support and care; staff were deeply concerned about service users who were experiencing reductions or withdrawals of care.

*Another personal pressure is I’m in this job because I want to help people and I’ve felt really like held back from that … the message at the beginning was don’t see clients, and I, kind of, really understood that. I’ve been taking the Covid really seriously in terms of like not going to supermarkets … but I felt really conflicted that we were leaving some very, very vulnerable clients without care.* (CBT therapist, Early intervention in psychosis service)

*That’s one of the things that this job isn’t about just medical stuff, it isn’t just about care coordination, it’s about recovery, relationships, hope, building something, and that’s a lot harder at the minute.* (Senior care coordinator, Early intervention in psychosis service)

Staff reported high levels of worry about service users who were not able to receive the care they needed. For example, staff in inpatient settings were anxious about their decisions to discharge service users into already stretched community services, and feared service users would deteriorate or in the worst case, die by suicide. In the event of the tragic news of a service user dying by suicide, staff reported a guilt and self-doubt about their decisions to discharge service users.

*I think it's important to say that we had a suicide of a patient a month after he was discharged. And so it was really tricky and I felt … I think it's tricky because you start kind of reviewing your decisions. I know it was a month before … it was a month after he was discharged so a lot of things could have happened, but you always question, like, was he really ready, were things in the community really ready for this kind of risk management.* (Trainee psychiatrist, Inpatient ward)

Many participants reported personal efforts to mitigate the potentially harmful effects of changes in care provision. For example, several members of staff continued to contact service users who, assigned to the ‘low risk’ category, and had been told that they needed to proactively call services if they needed support. Staff also made the case for seeing service users who they perceived to be particularly vulnerable face-to-face rather than using remote technologies. These additional tasks led to staff taking on considerably more work, exacerbating feelings of burnout.

Other dilemmas focused on trying to balance the need to control infection risk with the need for human contact in inpatient settings. Staff explained the difficulties of providing therapy or conducting necessary assessments with service users whilst wearing PPE. Staff also raised concerns about being able to react to emergencies rapidly because of the time taken to don PPE, and even once they had they reported some service users were startled or distressed by their appearance. In some cases, for example, service users with Covid-19 who did not agree with self-isolation protocols were, nevertheless, confined to their rooms. These measures put pressure on service users as well as staff, at times resulting in reported increased levels of distress and aggression, possibly due to frustration. In some services, frontline staff raised concerns about whether this form of restriction was ethically or legally defensible as part of care; emergency ethics committees were convened to address these and other pandemic-related dilemmas. Again, evidence of moral injury was a feature of these accounts.

*There have been challenges with the face-to-face interviews. I think the PPE is one of these challenges. I think arriving in full PPE, you’re a bit like an alien or a person from a nuclear reactor or something, and I think it’s hard to build a rapport with that. So, I’ve taken to arriving and then donning my PPE after introducing myself from a distance, which has gone a lot better.* (Trainee psychiatrist, Older adults CMHT)

*One of my clients who I saw in crisis … was kicking off and I turned up in PPE and [the client] … didn’t recognise that it was me and I [changed] my hair … so I stood back … showed him it was me, yeah, and then put the mask back [on] … Which I’m probably not supposed to do, but I was far enough away, showed him it was me and then sort of approached and then he was like … why did you change your hair?* (Senior care coordinator, Early intervention in psychosis service)

Staff participants were faced with further dilemmas around how best to care for service users displaying Covid symptoms. In some cases, participants reported a view that transferring service users to Covid wards would not be conducive to their mental health. In these situations, some staff made exceptions for service users who were deemed as particularly vulnerable, and cared for them using barrier-nursing methods.

*We feared that if we transferred this patient to a different ward to staff that don’t know [them], that that would make [their] treatment worse, so it wouldn’t be ideal for [their] mental state to be transferred. Additionally, we thought we could manage barrier nursing because every patient has their own room, most patients have their own toilet as well and I think this patient did. So, it was okay if we told the patient look you’ve got to stay in your room, you’ve got to only use your toilet and before we enter the room we’re going to get gowned up and everything like that.* (Trainee psychiatrist, Older adult rehabilitation ward)

### Wellbeing and the use of support

Irrespective of work location (on site or at home), some staff reported severe deterioration in their mental wellbeing. In those working in inpatient settings stress related directly to acuity and the impact of Covid restrictions on service users and staff, while in community services staff worried about the whereabouts and wellbeing of service users, and whether their mental health was deteriorating. In some cases, staff had to take a period of leave due to the development, or exacerbation of their own mental health difficulties. Several participants, for example, reported the deeply traumatic experience of witnessing many service user deaths over a short time period with little time to grieve and process what had happened; some of our participants had had to take leave themselves, for this reason. Other staff worried about service users that they were not in contact with and often expressed feelings of guilt and helplessness.

*You’re just helping people and you just go on, and on, and on, and all the 12-hour shifts. Transferring unwell patients to the main hospital, then keeping them for one night, and then the back and forth. ( … ) Then death after death after death. It was heart-breaking. Heart-breaking. Something that I was really not ready for. I know nobody was ready for it but … You know, on a psychiatric ward when a patient dies you get a debrief, but with this we never got any debrief for any of the patients. ( … ) The day I was going off sick, I was on the ward and I just started crying. I don’t even remember why now. I think I just broke down finally.* (Trainee psychiatrist, Inpatient ward)

Redeployment was linked to a set of difficult practical challenges for clinical leads tasked with managing workforce issues, such as training and ‘upskilling’ staff, supervising transitions between different types of service, and supporting staff who were, understandably, anxious about new working practices. In some cases, communication around redeployment decisions was poor, with staff told they were expected to attend a different service at very short notice, sometimes as little as 24 h. Some individuals who were asked to move from community services to inpatient wards described themselves as terrified of working in a new setting as well as the risk of Covid-19 transmission; some declined to do it. Risk assessment approaches for redeployment were a cause of frustration, as they did not appear to take into account professionals’ own mental health.

*I’ve read the risk assessment form and there’s nothing about assessing the risk of people working in the frontline for mental health. Has anyone had any history of loss? Has anyone had any mental health issues in the past? ( … ) They just randomly picked … There were trainees that just stayed in the community and worked from home, and there were people that were told that you need to go tomorrow, off you go.* (Trainee psychiatrist, Inpatient ward)

While it was, in principle, possible to take time off or to request a transfer to a different service, many participants avoided doing so because they did not want to add to the pressures on team members. Some felt they were doing more than “just a job”, highlighting how the ‘NHS heroes’ narrative, along with existing societal norms, encouraged staff to see their role as a moral responsibility.

*On the one hand I could be teleported out of the job to something else, but that’s really going to compound things for the team, and probably compound things for me, because I’d probably still be here a couple of days a week, and then make it worse for the patients, ‘cause who’s there to cover it? I’m just going to burden another [colleague].* (Trainee Psychiatrist, Older adults CMHT)

*I was really toying with the idea of not staying in this job because it wasn’t good for me ( … ). But then I thought, I’m a humanitarian at heart, if I bail on a pandemic what am I doing really?* (Trainee Psychiatrist, Acute adult ward)

Similarly, some staff who had to shield for medical reasons reported feeling guilty and helpless, and experienced a deep sense of isolation.

*I feel isolated at home. Normally, I’m a useful member of staff who can go in and do my work to a high quality and I have a team around me, I have people to talk to, have a giggle* (Nurse, Acute adult ward)

Many participants explained that managers had been transparent about the support on offer and often sent regular emails with details on how to access support, including links to staff wellbeing information, anonymous helpline numbers, and access to online digital therapy platforms such as SilverCloud. Other coping strategies included attending mindfulness courses, some of which were provided by their place of work, whereas other participants paid for private provision. In some cases, staff were redeployed to other healthcare settings to offer support to other staff, but some encountered challenges in balancing the needs of the staff and the service users:

*A psychologist has already been redeployed … to try and stem that, but anything that takes you away from your role is detrimental to patients, so I think balancing the needs of the staff and the organisation and the patients that we serve could be an interesting one.* (Senior clinical psychologist, Older adults CMHT)

The experiences of trainee doctors varied and some felt they did not receive the requisite support to deal with the changes. Many trainee doctors had assumed greater responsibilities as a result of their senior colleagues having to work from home, falling ill or shielding. Staff struggled with these added pressures, especially when they were accompanied by service changes and an absence of clarity around best practice Covid-19 guidelines, particularly during the acute phase of the crisis.

Some participants highlighted that support for staff was signposted by their employers but was not necessarily easy to access due to increased workload pressures and time constraints. Others commented on how their ability to understand how colleagues were coping and offer support was hindered by remote working.

*There was lots of different links that were available to … kind of offer some support. I mean, this is a dreadful thing to say, but I guess, the people who could get that support were the people working from home, because we were too busy. We couldn’t do that, because of time.* (Nurse, Psychiatric liaison service)

*It’s trickier to supervise because I have trainees and you get much more of a sense of how they’re doing when you’re all in an office together. And similarly, usually part of my role is support through team wellbeing and you can pick up on that a lot more easily when you’re all together, it’s a bit more filtered at the moment.* (Senior clinical psychologist, Older adults CMHT)

Staff reported that being mindful of the widespread effects of the pandemic appeared to help them to cope with increased pressures. The pandemic was referred to as ‘a leveller’ by staff, service users and carers alike. In some cases, this affected the dynamic of the relationships, as service users were more inclined to check in on how staff were feeling. Some staff reported feeling grateful they were able to get out of the house whilst others were forced to remain at home. Gestures of appreciation from service users, coupled with appreciation from the public, also helped some participants to navigate their work during an extremely difficult time.

*I mean, obviously the illness was dreadful, but … for once, the NHS was acknowledged … so, please don’t think I’m, you know, making light of the actual pandemic but for once … and like at work we were made a sandwich each day and we … got free tea and coffee. Now, that all sounds silly but it makes a huge difference … And so, and that was great … I mean, and so that made me feel very proud, you know.* (Nurse, Psychiatry liaison service)

## Discussion

This study of 35 mental health workers in the English NHS confirms that mental healthcare staff were exposed to a range of extreme conditions associated with the Covid-19 pandemic, and adds insights into the nature and range of the challenges they faced. The response to the pandemic meant that services had to rapidly adapt to major incident mode at the same time as the needs of service users increased. Participants reported multiple forms of stress, ranging from trauma associated with Covid-related deaths of people for whom they cared, anxiety associated with unusual and highly demanding working conditions and new practices, fatigue, and moral injury. At the same time as these emotional and psychological burdens intensified, they experienced isolation and deprivation of usual forms of social and professional support. Though some of these challenges for staff are known in other settings, demonstrating them in a mental healthcare context and providing insight into the nature of the challenges is valuable in responding to the calls to recognise and respond to the impact of the pandemic on the healthcare workforce [18].

A striking feature of the mental healthcare workers we interviewed about the experiences of the pandemic was their reports distress, other psychological morbidity and features of burnout. The latter involves emotional exhaustion, feelings of cynicism and detachment from work, and a sense of low personal accomplishment [19]. Some of these negative impacts are similar to those seen in other areas of health and social care such as, for some, the experience of seeing many service users die from Covid-19. However, a distinctive feature of the mental healthcare context is the evidence in our study of how modern principles of high-quality healthcare such as enabling choice, shared decision-making and offering therapeutic interventions came to be negated.

An especially prominent finding of our study was the extent to which forms of moral injury dominated mental healthcare workers’ accounts of their experiences. The concept of occupational moral distress and moral injury was developed in the context of active military combat and emergency response services. It describes suffering felt when staff are involved in, witness, or fail to prevent acts that may not necessarily be of any personal threat but, nevertheless, transgress deeply held moral expectations of their role [20]. Participants in our study had to deal with a wide range of moral dilemmas about, for example, restraining or isolating vulnerable individuals to prevent viral infection or transmission, feeling that they were providing sub-standard mental healthcare, that the form of work they did was undervalued and hidden, and that service users might experience iatrogenic harm as a result of paused, withdrawn or otherwise reconfigured care.

These are worrying reports, consistent with a view of moral injury as “perceived violation of one’s own professional integrity and obligations and concurrent feeling of being constrained from taking the ethically appropriate action” [21]. In an attempt to mitigate the risk of causing service users harm, some staff made a choice to ‘bend the rules’ in order to address the needs of service users, often at their own cost in terms of worry about the consequence. Some staff spent considerable time undertaking additional work, for example by visiting service users in person to avoid violating the values underpinning their personal morals and professional practice. Staff also displayed signs of moral injury in cases where it was not possible to contact service users and carers. Many were concerned about the effects of the ‘digital divide’ on service users and carers who were not able to engage with mental health services remotely [22]. Feelings of guilt and uncertainty about how service users and carers were faring during this time contributed to prolonged stress and other symptoms of burnout.

Recent work using a broader moral-philosophical notion of moral injury identifies the need for forms of moral repair alongside established psychological interventions for mental health symptoms [23]. This would combine two approaches: a preventive, psychological approach for staff at risk of moral injury that reinforces social bonds, is alert to early signs of distress, and avoids medicalisation of uncomfortable responses to trauma; and a moral repair approach that seeks to re-establish a sense of moral equilibrium using acts of acknowledgement that involve deep listening, altered understanding, and mutually agreed reparative action [23].

In seeking to advance such an approach, our study is important in demonstrating the extent to which informal knowledge-sharing and ‘mutual monitoring’, which is known to be essential for maintaining safety in healthcare teams [24], was compromised. These forms of informal social supports may be especially difficult to replace or replicate in pandemic conditions. However, Schwartz rounds – multidisciplinary forums where staff convene to discuss their work – are rich in potential [25] and their role (perhaps using online methods) should be evaluated further. Participants highlighted that while formal support for staff was signposted by their employers, it may not be easy to use, suggesting that availability of support may not be enough on its own – and not accessing support that is nominally available can become a source of stress in its own right. Also requiring further exploration is the role of structures for facilitating shared understanding and sound, morally defensible decisions, such as ethics committees. These have been unusual in mental health settings thus far, but warrant deeper examination.

The strengths of our study lie in its capturing of experiences of a wide range of mental healthcare staff in real-time, during the first wave of the Covid-19 pandemic. It does have a number of limitations. We did not conduct any formal measurement or assessment of the mental health of participants, relying on their own qualitative narratives only. Our study was based in NHS secondary mental healthcare, but did not include all point-of-care staff such as occupational therapists, healthcare assistants and peer-support workers. We also did not recruit staff from the full mental health workforce that includes social workers, helpline staff administrators and volunteers, as well as GPs. Nevertheless, we believe that the pressures and psychological consequences of providing mental healthcare during the pandemic are likely to be similar across these sectors, such that our study is relevant within and beyond NHS mental healthcare but acknowledge that further work in these settings is required. Further, we were unable to distinguish between the experiences of staff from different social groups or investigate the moderating effects of race. The study was conducted during a particular period, and may not describe the full chronology of experiences during the pandemic; it may well be that the effects we have described have changed during the subsequent peaks of Covid-19 infection that were not necessarily foreseen during the period of this investigation.

## Conclusions

Those working in mental healthcare have been affected by multiple adverse experiences while living with and working through the exigencies of the Covid-19 pandemic. Their experiences resulted in grief, loneliness, psychological stress/distress, and moral injury. These findings suggest areas where improved support could usefully be targeted.

## Data Availability

Requests for anonymised data should be made to the authors.

## References

[1] Hall LH, Johnson J, Watt I, Tsipa A, O’Connor DB (2016). Healthcare staff wellbeing, burnout, and patient safety: a systematic review. PLoS One.

[2] Serrano-Ripoll MJ, Meneses-Echavez JF, Ricci-Cabello I, Fraile-Navarro D, Fiol-deRoque MA, Pastor-Moreno G (2020). Impact of viral epidemic outbreaks on mental health of healthcare workers: a rapid systematic review and meta-analysis. J Affect Disord.

[3] Maben J, Adams M, Peccei R, Murrells T, Robert G (2012). ‘Poppets and parcels’: the links between staff experience of work and acutely ill older peoples’ experience of hospital care. Int J Older People Nursing.

[4] West CP, Dyrbye LN, Shanafelt TD (2018). Physician burnout: contributors, consequences and solutions. J Intern Med.

[5] Johnson J, Hall LH, Berzins K, Baker J, Melling K, Thompson C (2018). Mental healthcare staff well-being and burnout: a narrative review of trends, causes, implications, and recommendations for future interventions. Int J Ment Health Nurs.

[6] Lamb D, Gnanapragasam S, Greenberg N, Bhundia R, Carr E, Hotopf M, et al. The psychosocial impact of the COVID-19 pandemic on 4,378 UK healthcare workers and ancillary staff: initial baseline data from a cohort study collected during the first wave of the pandemic. medRxiv. 2021. 10.1101/2021.01.21.20240887. Accessed 10 May 2021. 10.1136/oemed-2020-107276 PMC8245285 34183447

[7] Bell V, Wade D. Mental health of clinical staff working in high-risk epidemic and pandemic health emergencies a rapid review of the evidence and living meta-analysis. Soc Psychiatry Psychiatr Epidemiol. 2020;56:1–11. 10.1007/s00127-020-01990-x PMC7691696 33245379

[8] Greenberg N, Docherty M, Gnanapragasam S, Wessely S (2020). Managing mental health challenges faced by healthcare workers during covid-19 pandemic. BMJ.

[9] Galbraith N, Boyda D, McFeeters D, Hassan T. The mental health of doctors during the COVID-19 pandemic. BJPsych bulletin. 2021;45(2):93-7. 10.1192/bjb.2020.44 PMC7322151 32340645

[10] Liu Y, Chen H, Zhang N, Wang X, Fan Q, Zhang Y, Huang L, Hu B, Li M (2021). Anxiety and depression symptoms of medical staff under COVID-19 epidemic in China. J Affect Disord.

[11] Liberati E, Richards N, Parker J, Willars J, Scott D, Boydell N, Pinfold V, Martin G,Dixon-Woods M, Jones P. Remote care for mental health: qualitative study with service users, carers and staff during the COVID-19 pandemic. BMJ open. 2021;11(4):e049210. 10.1136/bmjopen-2021-049210 PMC8068948 33888531

[12] Pereira-Sanchez V, Adiukwu F, El Hayek S, Bytyçi DG, Gonzalez-Diaz JM, Kundadak GK (2020). COVID-19 effect on mental health: patients and workforce. Lancet Psychiatry.

[13] Youn SJ, Creed TA, Stirman SW, Marques L. Hidden inequalities: COVID-19’s impact on our mental health workforce. 2020. Available from https://adaa.org/learn-from-us/from-the-experts/blogposts/professional/hidden-inequalities-covid-19s-impact-our. Accessed 10 May 2021.

[14] Palinkas LA, Horwitz SM, Green CA, Wisdom JP, Duan N, Hoagwood K (2015). Purposeful sampling for qualitative data collection and analysis in mixed method implementation research. Admin Pol Ment Health.

[15] Malterud K, Siersma VD, Guassora AD (2016). Sample size in qualitative interview studies: guided by information power. Qual Health Res.

[16] Charmaz K (2006). Constructing grounded theory: a practical guide through qualitative analysis.

[17] O’Brien BC, Harris IB, Beckman TJ, Reed DA, Cook DA (2014). Standards for reporting qualitative research: a synthesis of recommendations. Acad Med.

[18] Hall H. The effect of the COVID-19 pandemic on healthcare workers' mental health. J Am Acad PAs. 2020;33(7):45-8. 10.1097/01.JAA.0000669772.78848.8c 32590533

[19] Han S, Shanafelt TD, Sinsky CA, Awad KM, Dyrbye LN, Fiscus LC (2019). Estimating the attributable cost of physician burnout in the United States. Ann Intern Med.

[20] Williamson V, Stevelink SAM, Greenberg N (2018). Occupational moral injury and mental health: systematic review and meta-analysis. Br J Psychiatry.

[21] Lamiani G, Borghi L, Argentero P (2015). When healthcare professionals cannot do the right thing: a systematic review of moral distress and its correlates. J Health Psychol.

[22] Greer B, Robotham D, Simblett S, Curtis H, Griffiths H, Wykes T (2019). Digital exclusion among mental health service users: qualitative investigation. J Med Internet Res.

[23] Shale SJBL. Moral injury and the COVID-19 pandemic: reframing what it is, who it affects and how care leaders can manage it. 2020:leader-2020-000295.

[24] Waring Justin J, Bishop S (2010). “Water cooler” learning: knowledge sharing at the clinical “backstage” and its contribution to patient safety. J Health Organ Manag.

[25] McCarthy I, Taylor C, Leamy M, Reynolds E, Maben J (2020). ‘We needed to talk about it’: the experience of sharing the emotional impact of health care work as a panellist in Schwartz center rounds® in the UK. J Health Serv Res Policy.

